# Interstitial Surgical Cavity Sizing Applicators for the Treatment of the Breast Lumpectomy Cavity with Intraoperative Radiation Therapy

**DOI:** 10.7759/cureus.3693

**Published:** 2018-12-05

**Authors:** Lee C Goddard, N. Patrik Brodin, Amar Basavatia, Maureen McEvoy, Sheldon Feldman, Jana Fox, Keyur J Mehta, Wolfgang A Tomé

**Affiliations:** 1 Radiation Oncology, Montefiore Medical Center/Albert Einstein College of Medicine, Bronx, USA; 2 Surgery, Montefiore Medical Center/Albert Einstein College of Medicine, Bronx, USA

**Keywords:** intrabeam, intraoperative radiotherapy, iort, breast conservation, breast radiotherapy

## Abstract

Surgical cavity sizing applicators were developed for utilization prior to intraoperative radiation therapy (IORT) of the breast lumpectomy cavity with the Zeiss INTRABEAM (Carl Zeiss Meditec AG, Jena, Germany) device. The use of these applicators minimizes the number of sterilizations of the treatment applicator, which is currently limited to 100 sterilizations per applicator. This maximizes the number of patients who can be treated with each applicator, resulting in cost savings for the treating institution.

## Introduction

The Montefiore Medical Center began treating breast cancer patients with the Zeiss INTRABEAM 600 (Carl Zeiss Meditec AG, Jena, Germany) device in January 2018.Intraoperative radiation therapy (IORT) allows for the treatment of various disease sites immediately following the surgical resection of the tumor. Utilizing low-energy photons, large doses can be delivered to the target, with a rapid dose fall off, minimizing the dose to any adjacent healthy tissue [1-2]. The IORT applicators are available in a number of configurations to allow for an optimal dose delivery to different sites [3]. Spherical applicators are commonly used for the treatment of the breast lumpectomy cavity following tumor excision. Spherical applicators ranging from 3.0 to 5.0 cm in diameter in 0.5-cm increments are used at our institution allowing the selection of the applicator that best fills the surgical cavity. The correct-size applicator should be chosen to ensure a good conformation to the cavity, minimizing any air cavities, resulting in an optimal dose distribution. The exact sizing of the cavity ultimately requires an applicator placement, and multiple applicators may need to be placed before the optimal size applicator is determined. The Zeiss IORT treatment applicators have a limited number of sterilizations per applicator, and hence, the placement of multiple applicators results in having to re-sterilize each applicator that was used for sizing, even if they were not used for treatment, resulting in a reduced number of treatments over an applicator’s lifetime.

The use of IORT for low-risk localized breast cancer has gained a lot of interest following the results of the randomized targeted intraoperative radiotherapy A (TARGIT-A) trial [4-5] as well as large institutional series showing similar results [6-7]. Studies have also shown IORT to be a cost-effective treatment for early-stage breast cancer [8]. With an increasing number of cases, the cost- and resource-saving potential from eliminating unnecessary applicator sterilizations become considerable.

## Materials and methods

**Figure 1:**
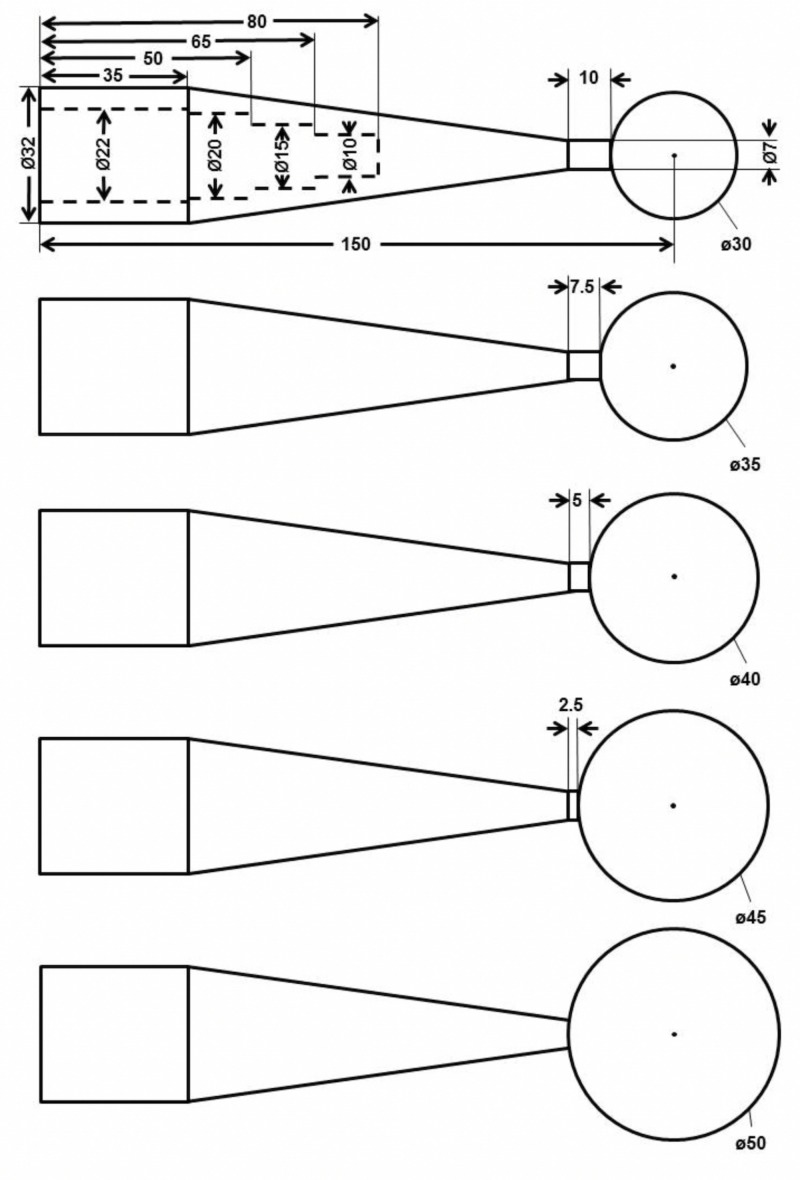
Schematic illustrating the dimensions of the MISCS applicators. All measurements are shown in millimeters. MISCS: Montefiore interstitial surgical cavity sizing

Montefiore interstitial surgical cavity sizing (MISCS) applicators that duplicate the size and shape of the treatment applicators were designed by the first and last authors and were manufactured by an external computer numerical controlled (CNC) machining service (QMC Technologies Inc., Depew, NY) using a surgical-grade stainless steel (the Society of Automotive Engineers [SAE] 316L), as shown in Figure 1. The cost of each applicator shown in Figure 1 was $700. These devices can be sterilized using high-temperature sterilization techniques with no limit on the number of sterilizations. The MISCS applicators can then be used to determine the exact size to be selected without the need for additional non-treatment usage of the applicators. At their relatively low cost, the MISCS applicators can save a significant number of sterilizations over the lifetime of each applicator, thereby ensuring the maximum treatment usage of each applicator (100 times). The sterilization process can also take a long time depending on the available facilities. The use of the treatment applicators to size the cavity can potentially result in delays while the applicators are sterilized. The MISCS applicators allow for potential time-saving in centers that are treating multiple patients daily and have limited numbers of treatment applicators.

## Results

**Figure 2:**
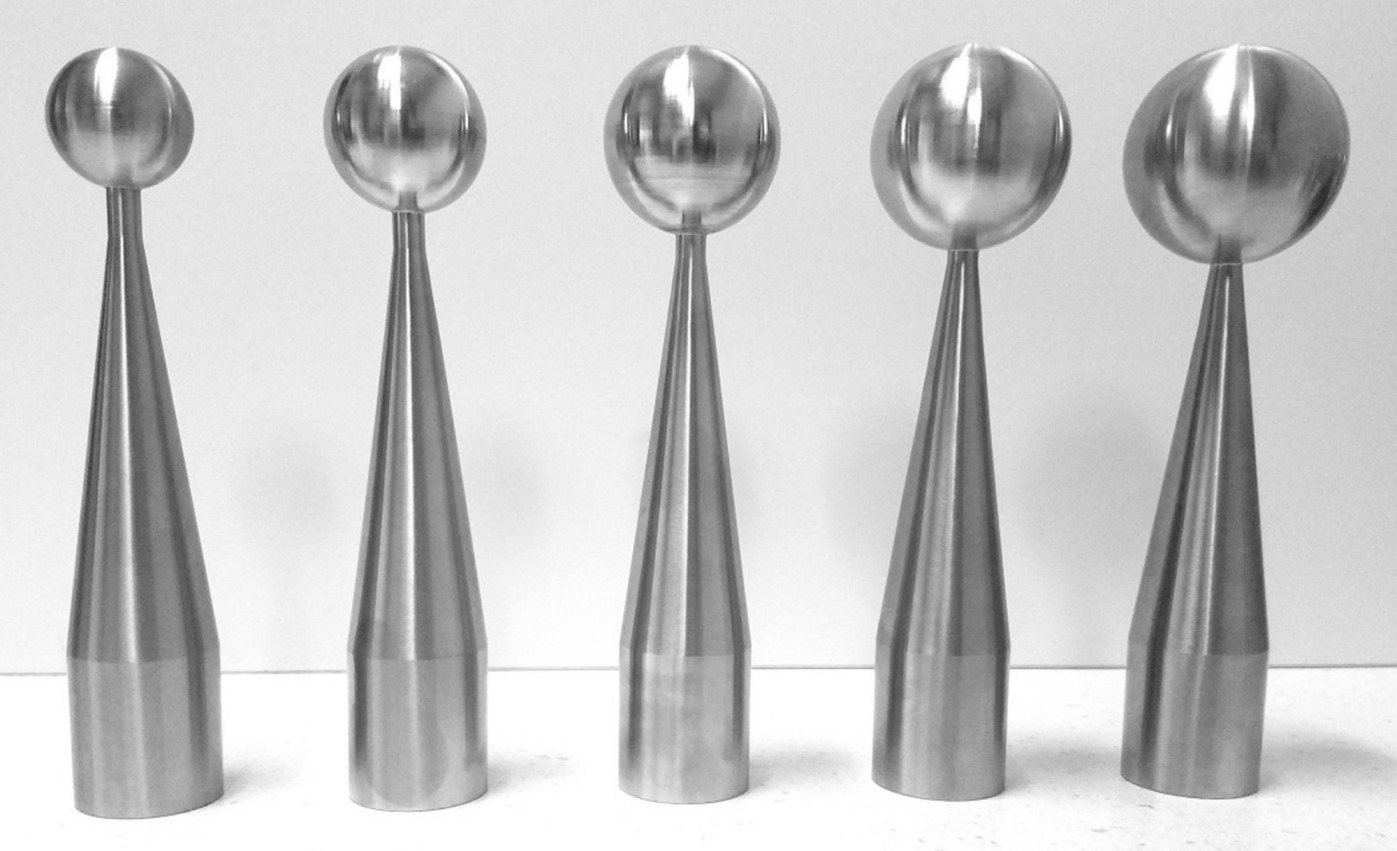
MISCS applicators, 3.0 to 5.0 cm diameter in 0.5-cm increments MISCS: Montefiore interstitial surgical cavity sizing

**Figure 3:**
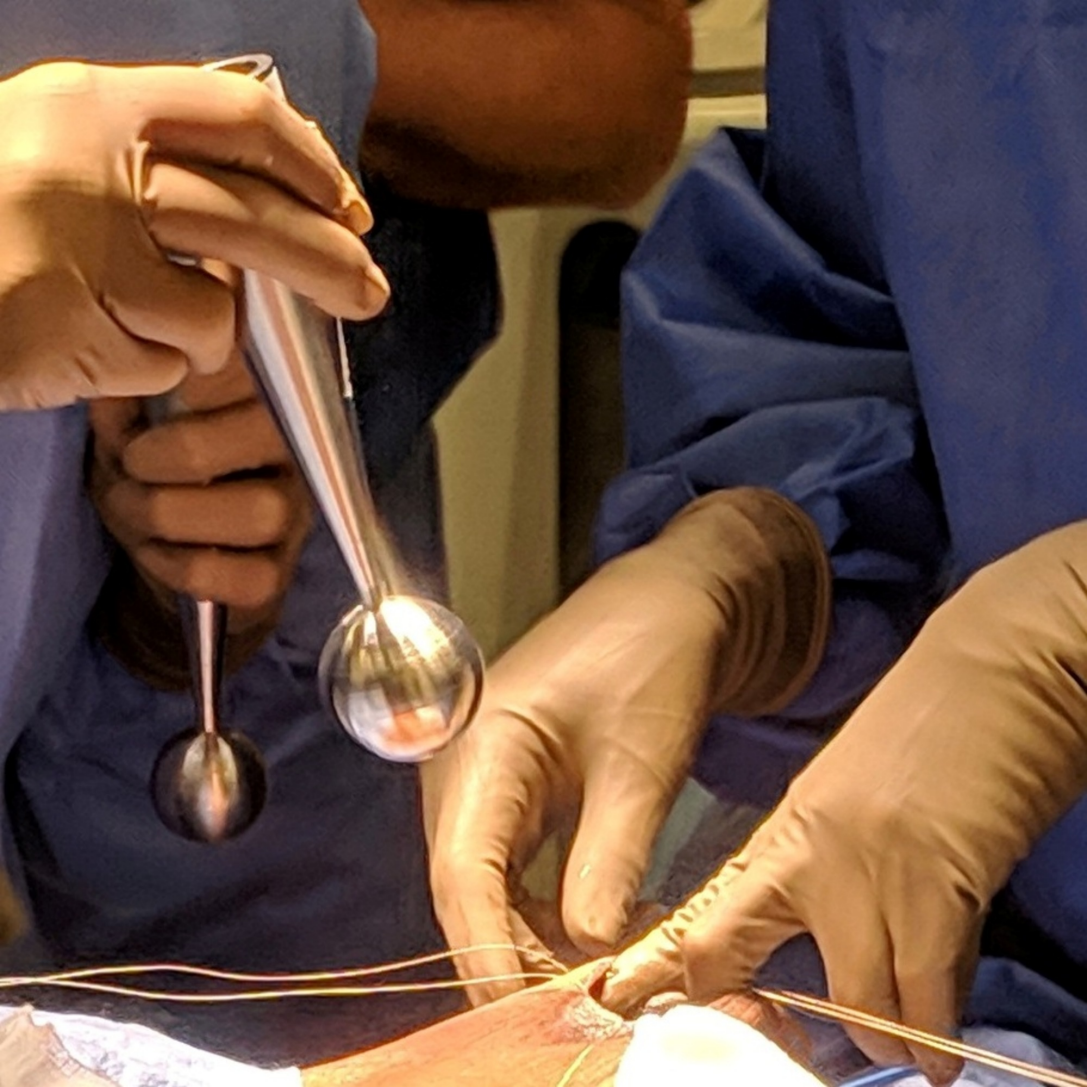
MISCS applicators being used prior to treatment to size the surgical cavity MISCS: Montefiore interstitial surgical cavity sizing

**Figure 4:**
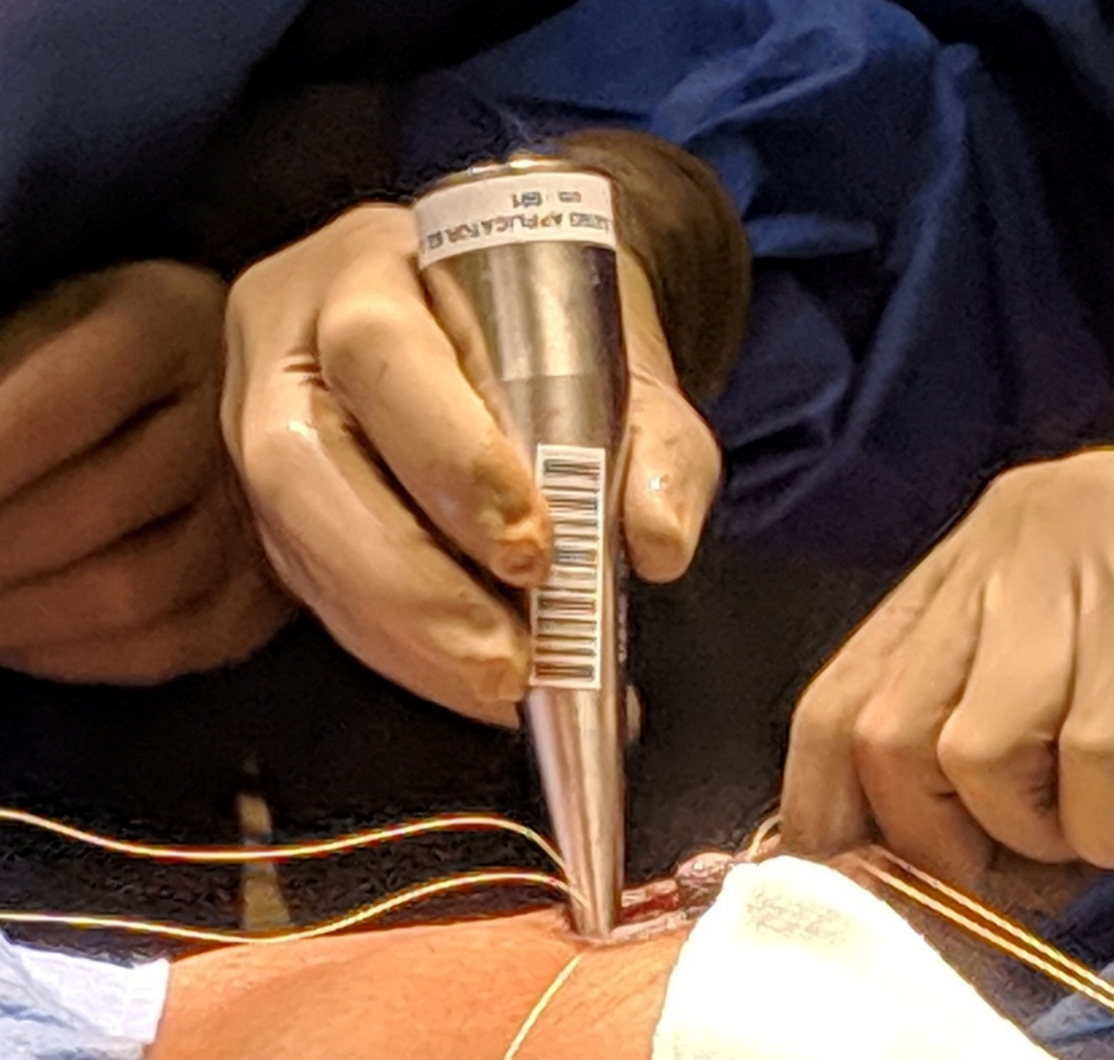
MISCS applicator inserted in the surgical cavity to check for correct fit MISCS: Montefiore interstitial surgical cavity sizing

The MISCS applicators (Figure 2) are stored in the sterilization trays with one applicator of each diameter, forming a set of five applicators. The Zeiss IORT treatment applicators are stored in individual sterilization trays. Upon completion of the lumpectomy, the sterile MISCS applicator set is opened, and the surgeon places the sizing applicator(s) within the cavity to determine the optimum applicator to be used for treatment (Figures 3-4).

## Discussion

Prior to the creation of the MISCS applicators, an average of two treatment applicators was utilized to size the surgical cavity for each case. This resulted in two sterilizations being required and effectively halving the number of cases treated over the lifetime of each applicator. Currently, treatment applicators are limited to 100 sterilizations per applicator. The cost of making a single set of MISCS applicators is approximately equal to the cost of a single treatment applicator. Thus, besides the ability to allow for an optimal cavity sizing through the use of as many applicators as needed with no regard to the need of re-sterilization, the MISCS applicators provide a financial benefit to the treating institution over the lifetime of a given treatment applicator set.

Furthermore, if the patient has undergone a bilateral lumpectomy, both cavities may be sized using one MISC set before treatment planning begins. This allows the physicist to create appropriate treatment plans and ensure only the corresponding size treatment applicators are opened.

Sterilization of the treatment applicators can also take a long time depending on the availability of sterilization equipment/personnel. By minimizing the excess sterilizations required, and having multiple sets of MISCS applicators available, the number of cases treated per day at a busy center can be increased and any delays to the patient and operating room schedule can be minimized.

## Conclusions

The MISCS applicators allow for the correct IORT treatment applicators to be selected, without requiring multiple treatment applicators to be utilized for each procedure. This results in maximizing the use and cost-effectiveness of each treatment applicator and optimizes patient throughput.
